# Positive Emotional Effects of Leisure in Green Spaces in Alleviating Work–Family Spillover in Working Mothers

**DOI:** 10.3390/ijerph14070757

**Published:** 2017-07-11

**Authors:** Po-Ju Chang, So Young Bae

**Affiliations:** 1 Program of Landscape and Recreation, National Chung Hsing University, Taiwan, No. 145 Hsing-Da Rd., Taichung 402, Taiwan; 2 College of Hotel and Tourism Management, Kyung Hee University, 26 Kyungheedae-ro, Dongdaemun-gu, Seoul 02447, Korea; soyoung.bae.20@gmail.com

**Keywords:** working mother, positive emotions, work-family spillover, family-work spillover, leisure in green spaces

## Abstract

Studies have shown that family and work spillover affects well-being and that leisure activities can alleviate the negative effects of work-related stress on health. However, few studies have focused on investigating the effects of specific leisure activities among specific populations. To examine whether leisure activities in green spaces can promote individual recovery processes and alleviate the effects of work and family spillover on positive emotions, this study applied the effort-recovery model to a population of working mothers. Through online and paper questionnaires, sample data were collected from 221 working mothers in Taiwan. Structural equation modeling was used to test the experimental hypothesis, and mediation analysis was used to determine whether leisure in green spaces is a mediating factor. The results indicated that leisure in green spaces is a mediator of the relationship of negative work and family spillover with positive emotions. In addition, strolls and park visits were found to provide greater psychological benefits to working mothers, compared with picnics.

## 1. Introduction

Per the 2014 report on Taiwanese women’s marriages, fertility, and employment [1], 55.9% of married women aged 15–64 years were employed. In Taiwanese families, women are usually the main caregivers, spending an average of 4.2 h every day on housework, four times the time that men spend. Therefore, the work–family balance for women has gradually become a concerning topic. Work and family experiences usually progress simultaneously, with working experiences affecting family life and vice versa. These interactive relationships shape and affect the health of the individual [2]: that is, the effects of family and work are bidirectional. Generally, in the interaction between family and work, emotions, feelings, stress, and behavior from one domain spill over and affect the other domain [3]. For example, work-induced stress can render an individual unable to participate in family activities, even after work. On the other hand, if family members fall sick, the burden of caring for the ill can cause an individual to lose concentration and become inefficient at work. These work–family interaction experiences are likely to have negative effects on the individual’s health [4].

Studies on work–family interactions have demonstrated that health intervention can assist individuals in managing their work and family life [5]. Leisure is considered a mediator in improving work and family balance. Participation in leisure activities is a health-promoting behavior that can moderate the relationship between stress and health [6]. Relevant studies on leisure have proven that participation in leisure activities can relieve work stress [7]. However, of the many types of leisure activities, those that have the greatest psychological benefit to working mothers remain unclear. Studies have established a relationship between green spaces and health. The socio–ecological framework on green spaces and health proposed by Lachowycz and Jones encompasses almost all factors associated with green spaces and health [8]. These authors indicated that participation in relaxing activities in green spaces, such as exercise, strolling, bird watching, chatting, and team activities, could produce positive physiological and health effects. Leisure activities in green spaces generate the greatest physical and health benefits. Therefore, this study examined whether participation in leisure activities in green spaces by working mothers can relieve the negative effects of work–family/family–work spillover on positive emotions.

### 1.1. Work–Family/Family–Work Spillover in Working Mothers

The main reason causing conflicts between work and family is “role conflict”. An individual plays many roles throughout the course of life. Women often play the roles of mother, employee, wife, and daughter. The responsibilities associated with these roles impose constraints on and induce stress in the individuals [9]; for example, a mother with two children at home may also be a senior manager at work. Studies have shown that work and family stress mutually interact, resulting in negative work–family and family–work spillovers. Negative work–family spillover has been found to be a chronic stressor, resulting in the individual producing psychological and physiological stress responses that damage health [2]. In addition, work–family conflict may cause individuals to develop a poor physiological and psychological health status [10]. For example, experiences of work–family conflict have been associated with depression symptoms [11], poor physiological health status, and the development of physiological health symptoms [12].

Work and family are two of the most important components of an individual’s life, but roles related to caring for the family are usually performed by women [13,14]. After women give birth to children, family responsibilities become heavier and more time-consuming. A study on the leisure patterns and constraints of urban women in Taiwan [15] argued that the leisure preference of women is associated with their life stage (i.e., age, age of the youngest child, and family composition), social achievements, and work conditions (i.e., education level, career, and working hours). Leisure participation by family members is the lowest when there is a 1- to 6-year-old child in the family [16]. Scholars have confirmed that women spend more time on all household labor than do men [17]. Similarly, marriage has a greater effect on the leisure activities of women than of men. Some scholars believe that the gender roles in a family are not only simply male and female roles but husband and wife roles: The wife, not the husband, is mainly responsible for household labor [18]. Thus, how to maintain a balance between work and family life is an important concern for employed women.

### 1.2. Effort-Recovery Model 

Although stress and constraints affect well-being, under some conditions, the individuals may spend time and effort on activities to improve their health [19]. The effort-recovery model provides a useful framework for researchers to examine how individuals decrease the negative effects of work–family/family–work spillover through their behaviors [20]. This model argues that the effort spent by an individual on work or non-work activities may eventually damage the individual’s health through a series of psychological, physiological, and behavioral processes. However, these processes are reversible. Individuals may reduce or reverse work-induced negative effects through participation in their favorite activities, as stress would gradually be released during the recovery process, thereby promoting physical health [21]. 

Studies have investigated how specific leisure activities, such as taking vacations [22], playing computer games [23], and participating in volunteer work [24], can enable an individual to recover positive resources. Van Hoff et al. [20] used the effort−recovery model to examine the relationship between work–family conflict and the daily activity pattern of employees and reported that employees who participated in more low-effort leisure-time physical activities were less likely to experience work–family conflicts. Lee et al. [25] investigated the role of leisure-time physical activities in work–family conflict and healthy long-term relationships in middle-aged subjects; they found that high participation in moderate leisure-time physical activities could reduce individual stress engendered by work–family imbalance. Therefore, the current study assumed that working mothers are busy with work or familial care activities and that they spend long hours indoors both for work and household activities. If they are freed from the environment of work and family activities, they may relax and experience stress relief. Specifically, participating in relaxing outdoor leisure activities may be the best method of relieving the negative effects of work–family/family–work spillover on the well-being of working mothers.

### 1.3. Leisure in Green Spaces

Studies have found that participation in outdoor leisure activities exposes individuals to the natural environment, resulting in great health benefits. Bratman, Hamilton, and Daily [26] summarized two theories to explain the contribution of the natural environment in relieving stress: stress recovery theory and attention restoration theory. Stress recovery theory proposed by Ulrich states that the natural environment can enhance the positive emotions of an individual through visual characteristics (e.g., the wider perspective), which can help relieve stress [27]. Attention restoration theory presented by Kaplan and Kaplan states that the natural environment can ameliorate attention fatigue and relieve stress, thereby restoring direction attention [28]. Shi, Gou, and Chen [29] examined the relationships between urban residents’ environmental preferences and enclosure of urban public open spaces in Hong Kong, and they reported that participants preferred open spaces, which enabled them to relieve their daily stress. Hartig and Staats found that strolling in urban parks not only promotes positive emotions but also decreases negative emotions [30]. Weng and Chiang compared indoor leisure activities and leisure activities in natural environments [31]; they found that among 203 Taiwanese subjects, individuals who participated more in leisure activities in green spaces, such as strolling and exercises in urban green spaces, reported lower levels of anxiety and higher restoration of attention. In other words, different leisure activities in green spaces may contribute differently to stress restoration. For example, Lau, Gou, and Liu [32] summarized the health effects of different types of natural views (e.g., trees, various degrees of nature in window views, gardens, and park-like forest areas with creek) and concluded that green plants, flowers, and water are the most beneficial natural elements that foster stress restoration. Furthermore, individuals’ casual interactions with these natural elements (e.g., walking through green open spaces) could relax their tense nerves and relieve their stress. In addition, the authors categorized leisure activities in green open spaces as transitional (e.g., passing-by), personal (e.g., taking a walk), and social (e.g., hanging out with friends) activities and suggested that the best restorative natural environment is one that allows users to easily engage in personal activities and social interactions. 

Lachowycz and Jones developed a social–ecological framework on green spaces and health based on the relevant literature and divided the resulting health benefits into two major categories: physical health and psychological health [8]. Physical health benefits are usually associated with physical activities in green spaces, whereas psychological health benefits are derived from contact with natural elements and through social interactions. In addition, the theoretical framework proposed by Lachowycz and Jones includes potential coordination factors in the green space–health relationship, such as age, gender, social background, and profession. The outdoor setting of leisure in green spaces may provide an environment that is very different from the indoor working or family environment experienced by working mothers. This enables working mothers to experience one of the main benefits of leisure: escape from stress in daily life.

### 1.4. Summary

The main objective of this study was to develop the effort-recovery model and the social-ecological framework of green space and health into a theoretical framework. This framework was used to examine the relationship between family and work conflict, leisure activities in green spaces, and positive emotions in working mothers. The two main research questions are outlined as follows: (1) Do family and work conflicts affect the positive emotions of working mothers? (2) Can leisure activities in green spaces mediate the negative effects of work–family/family–work conflicts on positive emotions? To answer these research questions, this study employed online and paper questionnaires in 2017 to collect relevant data from working mothers in Taiwan.

## 2. Materials and Methods

### 2.1. Samples

From February to April 2017, data were collected through paper and online questionnaires administered to urban employed women with at least one child. The paper questionnaires were distributed in Taichung, the largest city in central Taiwan, which has 13 public greenways and many green open spaces for urban residents to involve in outdoor leisure activities. Taichung is also scheduled to host the 2018 World Flora Exposition. The online questionnaires were distributed through Qualtrics (www.qualtrics.com), a website that enables users to create questionnaires and collect responses online; the URL link to the questionnaire was posted on online communities for Taiwanese mothers residing in Taiwan. Overall, 243 women completed the paper and online questionnaires. Before distributing the questionnaire, we explained the purpose of the study, the content of the questionnaire, and concerns regarding responder anonymity. All participants agreed and gave their informed consent for their inclusion in the study. The questionnaires included questions on work–family/family–work spillover, frequency of participation in leisure activities in green spaces, and positive emotions. To ensure accuracy, only those questionnaires in which all three sections were completed were included in the analysis; this approach yielded 221 final questionnaires. The participants were mainly women aged 30–39 years (68.3%), and most only had one child (63.3%) (Table 1). More than half of the participants had 4-year university education level and above (74.7%). Monthly household income was roughly evenly distributed in each category, with a slight preponderance (24.4%) in the NT$60,000–NT$80,000 (US$1994–US$2658) category.

### 2.2. Measurement Scale

The survey scales measured work–family conflict, frequency of participation in leisure activities in green spaces, positive emotions, and demographic information, as explained herein.

#### 2.2.1. Work–Family Conflict

The work and family scale of the Midlife Development in the United States (MIDUS) study, developed by Grzywacz and Marks [2], was used for the work–family conflict scales. The original scale contained four spillover subscales: positive work–family spillover, positive family–work spillover, negative work–family spillover, and negative family–work spillover. In line with the study objectives, this study used the two negative spillover scales: the negative work–family spillover scale, which covers such topics as “Does your work decrease the number of family activities you participate in?”, and the negative family–work spillover scale, which covers such topics as “Does your family responsibilities decrease your efforts during work?” The two scales each contain four questions, which are responded to on a 5-point Likert scale (1 = *strongly disagree* to 5 = *strongly agree*). The reliability (Cronbach’s alpha) levels of the negative work–family and family–work spillover scales were measured to be 0.842 and 0.766, respectively.

#### 2.2.2. Frequency of Participation in Leisure Activities in Green Spaces

The degree of participation in leisure activities in green spaces scale contains three items on leisure activities involving contact with the natural environment, responded to on a 5-point Likert scale (1 = never to 5 = daily). Activities in green spaces include park visits, picnics, and strolls. The reliability (Cronbach’s alpha) of this scale was measured to be 0.685.

#### 2.2.3. Positive Emotions

The positive emotion scale of the PERMA model, developed by Seligman, was used as the positive emotion scale [33]. It consists of three topics, responded to on a 5-point Likert scale (1 = strongly disagree to 5 = strongly agree), which cover such items as “Generally, I am happy with my current life.” The reliability (Cronbach’s alpha) of this scale was measured to be 0.853.

#### 2.2.4. Demographic Information

Through a literature review, we identified that work–family conflicts, participation in leisure activities, and emotions in working mothers were all related to individual social and financial characteristics. Therefore, we collected data on education level (1 = high school, 2 = 2-year technical college, 3 = 4-year university, and 4 = graduate school) and monthly household income (1 = less than NT$40,000 or US$1329 to 6 = more than NT$120,000 or US$3986) as control variables.

### 2.3. Analysis

**Figure 1:**
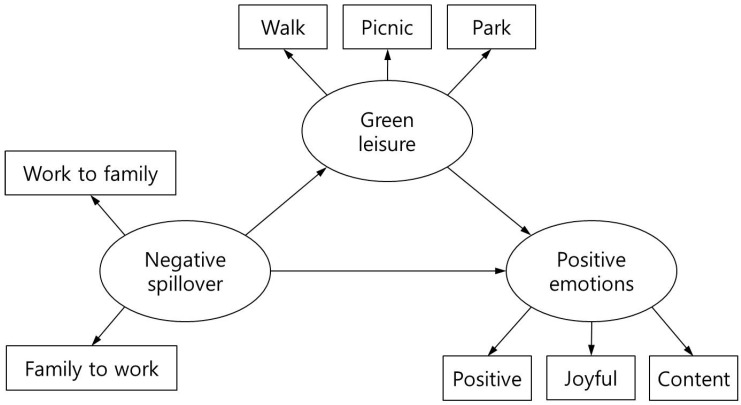
Tested conceptual model.

This study conducted structural equation modeling of all data by using SPSS AMOS to analyze whether “leisure activities in green spaces” contributed to the relationship between “negative work–family/family–work spillover” and “positive emotions,” after controlling for education level and monthly household income. After the establishment of the hypothesized model, the mediation model conditions proposed by Baron and Kenny were used to verify the mediation model of “negative work–family/family–work spillover”—“leisure activities in green spaces”—“positive emotions” (Figure 1). Baron and Kenny proposed a four-step verification method for the mediation model [34]: (1) the predictor variable “negative work–family/family–work spillover” and the result variable “positive emotions” show a significant relationship; (2) the predictor variable “negative work–family/family–work spillover” and the mediator variable “leisure activities in green spaces” show a significant relationship; (3) the mediator variable “leisure activities in green spaces” and the result variable “positive emotions” show a significant relationship; (4) after addition of the mediator variable, the effect of the predictor variable on the result variable is reduced.

## 3. Results

### 3.1. Descriptive Statistics

All variables exhibited mutual relationships (Table 2), which is consistent with the literature. For example, leisure activities in green spaces were negatively correlated with both items of negative spillover (i.e., negative work–family spillover and family–work spillover), and when an individual experiences more negative spillover between work and family, the individual’s participation in leisure activities in green spaces is lower, and vice versa. In addition, negative spillover and positive emotions were negatively correlated, meaning that if an individual experiences a higher level of negative spillover between work and family, the individual experiences a lower level of positive emotions. Moreover, the frequency of participation in leisure activities in green spaces exhibited a positive correlation with positive emotions; in other words, individuals who participate in more leisure activities in green spaces experience more positive emotions.

### 3.2. Verification of Baron and Kenny’s Mediation Model 

Our model fulfilled Baron and Kenny’s mediation model conditions (Table 3); that is, all routes were significant, and the effect of “negative spillover” on “positive emotions” was reduced when the mediator variable “leisure activities in green spaces” was added. Thus, our results verified that leisure activities in green spaces exert a mediating effect in the relationship between negative spillover and positive emotions.

### 3.3. SEM Assessment

**Figure 2:**
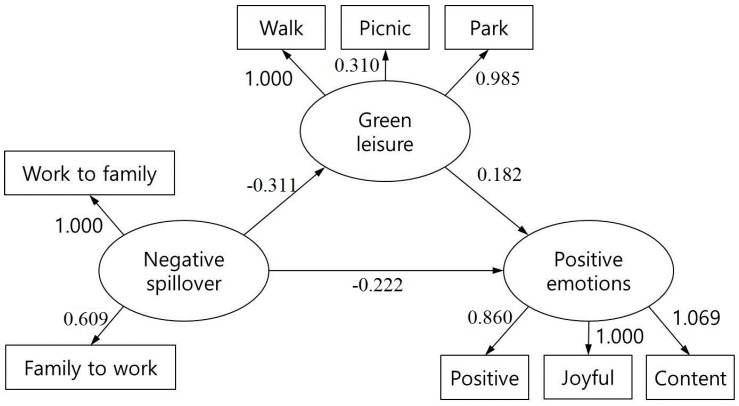
Final model in the current study. All paths significant at the *p* < 0.05 level with RESMA = 0.019, TLI = 0.964, and CFI = 0.982. Note that we controlled for education and financial status.

Our results support the hypothetical mediation model of “negative work–family/family–work spillover”—“leisure activities in green spaces”—“positive emotions” (Figure 2). The model goodness-of-fit indicators—root mean square error of approximation 0.019 < 0.080, Tucker-Lewis index 0.964, and comparative fit index 0.982—all satisfied the model fit criteria, and the hypothetical model agreed with the sample data. “Leisure activities in green spaces” was verified to be a mediator between “negative spillover” and “positive emotions,” meaning that leisure activities in green spaces effectively relieve the negative effects of negative work–family/family–work spillover among working mothers on positive emotions. Participation in leisure activities in green spaces ameliorates the negative spillover of work and family in working mothers, thereby affecting their positive emotions. Furthermore, work–family conflict (standardized β = 1.000) was greater than family–work conflict (standardized β = 0.609), and strolls and park visits in green spaces were found to make relatively larger contributions to this process (standardized β = 1.000 and 0.985, respectively). For positive emotions, “feeling that life is enriched” was the factor with the highest contribution value (standardized β = 1.069).

## 4. Discussion

Our results confirm the exposition of the “effort-recovery model” and the “social-ecological framework of green spaces and health.” Work–family conflict reduced the positive emotions of working mothers, and mediation by leisure activities in green spaces can improve this relationship. When working mothers cannot balance work and family activities, which transfers the stress from one life domain to another, they can go outdoors and interact with nature through leisure activities, and temporarily leave their stressors (i.e., the indoor environment). By immersing in a natural environment that is different from their workplace or family environment, they can relieve stress and increase their positive emotions.

In work–family conflict, work stress faced by working mothers exerts greater negative effects on family. Working mothers are required to care for their families and simultaneously manage responsibilities at work. The 2015 Taiwanese Women Awakening Foundation survey on “Childbearing-friendly workplace” [35] indicated that Taiwanese female workers were often discriminated against at their workplace due to pregnancy and that they faced difficulties in applying for childcare leave. An unfriendly work environment can induce additional stress in working mothers, further affecting their family life. Stress and constraints that spill over from the work domain into family life can directly affect the positive emotions of working mothers. Using Baron and Kenny’s mediation model conditions, we confirmed that leisure activities in green spaces could alleviate the effects of work–family conflict on positive emotions in working mothers. Per stress recovery theory and attention restoration theory, spending time in a natural environment can relieve stress [26,27]. Leisure in green spaces provides working mothers an opportunity to spend time in a natural environment to relieve stress. Moreover, per the effort-recovery model, the negative effect of work–family spillover might be a trigger for working mothers to engage in healthy behavior [19,20] (e.g., leisure activities in green spaces) in order to release their stress during the recovery process. 

In the category of leisure activities in green spaces, strolls and park visits can better alleviate work–family conflict in working mothers than do picnics. Going on a picnic may be viewed as a form of family leisure. In other words, the setting of this leisure activity is still in the family domain. In a family picnic, the responsibilities of preparing the food and itinerary are usually borne by the women in the family, who are usually the main caregiver in a Taiwanese family. Additionally, they must take care of children during picnics. These reasons may be why mothers cannot relax and enjoy such outdoor leisure activities. Therefore, to working mothers, picnics may not be a form of leisure activity that can relieve stress. By contrast, strolls and park visits do not require preparation beforehand and can be done by oneself. Working mothers can stroll in parks to relieve stress due to an imbalance in work and family life and fully enjoy the environment of green spaces. Although research suggests that personal activities and social interactions in natural environments could greatly restore attention [35], the best type of leisure in green spaces for working mothers may be personal activities that are not related to their work or family domain.

This study makes academic and practical contributions. Our study combined the theories of the effort-recovery model and social-ecological framework on green spaces and health and can practically be applied to the population of working mothers in Taiwan. We investigated specific leisure settings such as green spaces. Subsequent studies could compare employed and unemployed mothers and focus on more types of green spaces. Regarding practical contributions, the study results can be shared with relevant work institutions. Stress in working mothers could be relieved by providing green spaces in the workplace or through landscape therapy and other interventions, thereby promoting work efficiency. The 2016 fertility rate in Taiwan was 1.2, the lowest in the world [36]. Consequently, the older population in Taiwan is increasing rapidly beyond 12%, making Taiwan an aging society [37]. When the work environment and atmosphere are friendly for women, becoming a working mother and having more than one child might not be viewed as a disadvantage in Taiwan, which might reduce the rate of decrease in fertility. 

## 5. Conclusions

This study was based on the effort-recovery model and green-health model and highlights the contributions of leisure activities in green spaces in the effects of work–family conflicts on positive emotions. Strengthening the understanding of effects of green spaces and health on this population is important for promoting and maintaining the well-being of working mothers. Through the use of our results as a basis, effective health intervention programs can be provided. Our study confirmed that relaxing leisure activities in green spaces best relieve work–family conflict in working mothers. Subsequent studies and practitioners can consider the factor of leisure activities in green spaces to develop relevant policies on workplace health.

## Figures and Tables

**Table 1:** Demographic information of study sample.

Variables	Frequency (%)
Age	
20–29	34 (15.4)
30–39	159 (68.3)
40–49	18 (8.2)
Over 50	10 (4.5)
Number of children	
1	140 (63.3)
2	66 (29.9)
3 or more	15 (6.8)
Education	
High school	17 (7.7)
2-year college	39 (17.6)
University	97 (43.9)
Graduate school	68 (30.8)
Monthly household income (TWD *)	
<40,000	26 (11.8)
40,000–60,000	33 (14.9)
60,000–80,000	54 (24.4)
80,000–100,000	47 (21.3)
100,000–120,000	23 (10.4)
>120,000	38 (17.2)

* US$ 1 = 30.36 TWD (Taiwan dollar).

**Table 2:** Correlation coefficients of the study variables.

Variable	1	2	3	4
1. Negative W-F spillover				
2. Negative F-W spillover	0.444 **			
3. Green leisure	−0.292 **	−0.147 *		
4. Positive emotions	−0.035 *	−0.086 *	0.117 *	
Mean	3.326	3.199	2.409	3.611
SD	0.903	0.800	0.665	0.798

* *p* < 0.05. ** *p* < 0.01.

**Table 3:** Modified Path Model and Test of the Mediating Effect.

Path	Standardized β (SE)
First condition	
Negative spillover → Positive emotions	−0.325 (0.118)
Second condition	
Positive emotions → Leisure in green spaces	−0.246 (0.141)
Third condition	
Leisure in green spaces → Positive emotions	0.258 (0.004)
Fourth condition	
Negative spillover → Positive emotions	−0.222 (0.103)

Note. All paths significant at the *p* < 0.05 level.

## References

[1] (2014). The 2014 Report on Women’s Marriages, Fertility, and Employment in Taiwan.

[2] Grzywacz G., Marks N.F. (2000). Family, work, work–family spillover, and problem drinking during midlife. J. Marriage Fam..

[3] Mennino S.F., Rubin B.A., Brayfield A. (2005). Home-to-job and job-to-home spillover: The impact of company policies and workplace culture. Sociol. Q..

[4] Greenhaus J.H., Powell G.N. (2006). When work and family are allies: A theory of work–family enrichment. Acad. Manag. Rev..

[5] Hammer L.B., Kossek E.E., Anger W.K., Bodner T., Zimmerman K.L. (2011). Clarifying work–family intervention processes: The roles of work–family conflict and family-supportive supervisor behaviors. J. Appl. Psychol..

[6] Qian X., Yarnal C., Almeida D.M. (2013). Does leisure time as a stress coping resource increase affective complexity? Applying the Dynamic Model of Affect (DMA). J. Leis. Res..

[7] Nimrod G., Kleiber D.A., Berdychevsky L. (2012). Leisure in coping with depression. J. Leis. Res..

[8] Lachowycz K., Jones A.P. (2013). Towards a better understanding of the relationship between greenspace and health: Development of a theoretical framework. Landsc. Urban Plan..

[9] Greenhaus J.H., Beutell N.J. (1985). Sources of conflict between work and family roles. Acad. Manag. Rev..

[10] Kim S., Okechukwu C.A., Buxton O.M., Dennerlein J.T., Boden L.I., Hashimoto D.M., Sorensen G. (2013). Association between work-family conflict and musculoskeletal pain among hospital patient care workers. Am. J. Ind. Med..

[11] Jawahar I.M., Kisamore J.L., Stone T.H., Rahn D.L. (2012). Differential effect of inter-role conflict on proactive individual’s experience of burnout. J. Bus. Psychol..

[12] Amstad F.T., Meier L.L., Fasel U., Elfering A., Semmer N.K. (2011). A meta-analysis of work-family conflict and various outcomes with a special emphasis on cross-domain versus matching-domain relations. J. Occup. Health Psychol..

[13] Coltrane S. (2000). Research on household labor: Modeling and measuring the social embeddedness of routine family work. J. Marriage Fam..

[14] Hook J. (2006). Care in context: Men’s unpaid work in 20 countries, 1965–2003. Am. Sociol. Rev..

[15] Lee S.H. (1997). Urban women leisure patterns and constraints. J. Outdoor Recreat. Study.

[16] Hsieh S.F. (2003). A Study on the relationships among gender, family life cycle, family leisure participation frequency, and leisure constraints. Tour. Manag. Res..

[17] Sanchez L., Thomson E. (1997). Becoming mothers and fathers: Parenthood, gender, and the division of labor. Gend. Soc..

[18] West C., Zimmerman D.H. (1987). Doing gender. Gend. Soc..

[19] Bordin E.S., Savikas M.L., Lent R.W. (1994). Intrinsic motivation and the active self: Convergence from a psychodynamic perspective. Convergence in Career Development Theories: Implications for Science and Practice.

[20] Van Hooff M., Geurts S.A.E., Kompier M.A.J., Taris T.W. (2006). Work-home interference: How does it manifest itself from day to day?. Work Stress.

[21] Sonnentag S. (2001). Work, recovery activities, and individual well-being: A diary study. J. Occup. Health Psychol..

[22] Eden D., Cooper C, Robertson I.T. (2001). Vacations and other respites: Studying stress on and off the job. Well-Being in Organizations.

[23] Reinecke L. (2009). Game at work: The recreational use of computer games during working hours. Cyberpsychol. Behav..

[24] Mojza E.J., Lorenz C., Sonnentag S., Binnewies C. (2010). Daily recovery experiences: The role of volunteer work during leisure time. J. Occup. Health Psychol..

[25] Lee B., Lawson K.M., Chang P.J., Neuendorf C., Dmitrieva N.O., Almeida D.M. (2015). Leisure-time physical activity moderates the longitudinal associations between work-family spillover and physical health. J. Leis. Res..

[26] Bratman G.N., Hamilton J.P., Hann K.S., Daily G.C., Gross J.J. (2012). Nature experience reduces rumination and subgenual prefrontal cortex activation. Proc. Natl. Acad. Sci. USA.

[27] Ulrich R.S. (1983). Aesthetic and affective response to natural environment. Behav. Nat. Environ..

[28] Kaplan R., Kaplan S. (1989). The Experience of Nature: A Psychological Perspective.

[29] Shi S., Gou Z., Chen L.H.C. (2014). How does enclosure influence environmental preferences? A cognitive study on urban public open spaces in Hong Kong. Sustain. Cities Soc..

[30] Hartig T., Henk S. (2003). Guest editors’ introduction: Restorative environments. J. Environ. Psychol..

[31] Weng P.Y., Chiang Y.C. (2014). Psychological restoration through indoor and outdoor leisure activities. J. Leis. Res..

[32] Lau S.S.Y., Gou Z., Liu Y. (2014). Healthy campus by open space design: Approaches and guidelines. Front. Archit. Res..

[33] Seligman M. (2011). Flourish: A Visionary New Understanding of Happiness and Well-Being.

[34] Baron R., Kenny D.A. (1986). The moderator-mediator variable distinction in social psychological research: Conceptual, strategic, and statistical considerations. J. Pers. Soc. Psychol..

[35] The Taiwanese Women Awakening Foundation (2015). The 2015 Childbearing-Friendly Workplace Survey.

[36] Population Reference Bureau. www.prb.org.

[37] National Development Council Population Projections for Republic of China (Taiwan): 2014–2060. www.ndc.gov.tw.

